# Cell separation in kiwifruit without development of a specialised detachment zone

**DOI:** 10.1186/s12870-017-1034-2

**Published:** 2017-05-10

**Authors:** Roneel Prakash, Ian C. Hallett, Sally F. Wong, Sarah L. Johnston, Erin M. O’Donoghue, Peter A. McAtee, Alan G. Seal, Ross G. Atkinson, Roswitha Schröder

**Affiliations:** 1 The New Zealand Institute for Plant & Food Research Limited (PFR), Mount Albert Research Centre, Private Bag 92169, Auckland, 1142 New Zealand; 2 PFR, Mount Albert Research Centre, Private Bag 92169, Auckland, 1142 New Zealand; 3 PFR, Hawke’s Bay Research Centre, Cnr Crosses and St George’s Roads, Havelock North, 4130 New Zealand; 4 PFR, Food Industry Science Centre, Fitzherbert Science Centre, Batchelar Road, Palmerston North, 4474 New Zealand; 5 PFR, Te Puke Research Centre, 412 No 1 Road RD 2, Te Puke, 3182 New Zealand

**Keywords:** Cell wall, Detachment, Kiwifruit, Peeling, Polygalacturonase, Transglycosylase

## Abstract

**Background:**

Unlike in abscission or dehiscence, fruit of kiwifruit *Actinidia eriantha* develop the ability for peel detachment when they are ripe and soft in the absence of a morphologically identifiable abscission zone. Two closely-related genotypes with contrasting detachment behaviour have been identified. The ‘good-peeling’ genotype has detachment with clean debonding of cells, and a peel tissue that does not tear. The ‘poor-peeling’ genotype has poor detachability, with cells that rupture upon debonding, and peel tissue that fragments easily.

**Results:**

Structural studies indicated that peel detachability in both genotypes occurred in the outer pericarp beneath the hypodermis. Immunolabelling showed differences in methylesterification of pectin, where the interface of labelling coincided with the location of detachment in the good-peeling genotype, whereas in the poor-peeling genotype, no such interface existed. This zone of difference in methylesterification was enhanced by differential cell wall changes between the peel and outer pericarp tissue. Although both genotypes expressed two polygalacturonase genes, no enzyme activity was detected in the good-peeling genotype, suggesting limited pectin breakdown, keeping cell walls strong without tearing or fragmentation of the peel and flesh upon detachment. Differences in location and amounts of wall-stiffening galactan in the peel of the good-peeling genotype possibly contributed to this phenotype. Hemicellulose-acting transglycosylases were more active in the good-peeling genotype, suggesting an influence on peel flexibility by remodelling their substrates during development of detachability. High xyloglucanase activity in the peel of the good-peeling genotype may contribute by having a strengthening effect on the cellulose-xyloglucan network.

**Conclusions:**

In fruit of *A. eriantha,* peel detachability is due to the establishment of a zone of discontinuity created by differential cell wall changes in peel and outer pericarp tissues that lead to changes in mechanical properties of the peel. During ripening, the peel becomes flexible and the cells continue to adhere strongly to each other, preventing breakage, whereas the underlying outer pericarp loses cell wall strength as softening proceeds. Together these results reveal a novel and interesting mechanism for enabling cell separation.

**Electronic supplementary material:**

The online version of this article (doi:10.1186/s12870-017-1034-2) contains supplementary material, which is available to authorized users.

## Background

During plant cell separation, the polysaccharide networks of the cell wall that connect cells are dismantled with ‘surgical precision’ [1]. Cell separation can lead to total detachment either within an organ (dehiscence) or between an organ and the parent plant (abscission). Cell separation can also occur locally, leading to development of intercellular spaces in leaves or fruit for example. Generally, both abscission and dehiscence take place at predetermined zones of isodiametrically flattened cells, arranged in anything between two to 50 cell layers [2, 3]. These cells start to differentiate some time before the actual organ separation, and remain arrested in this developmental stage until a diverse set of signals, including ethylene and auxin, trigger a cascade of expression of genes whose products regulate the actual cell separation. In the model that Sexton and Roberts [4] presented, ethylene is the primary signal that drives the leaf abscission process, whereas auxin reduces the sensitivity of abscission zone cells to ethylene, thus preventing or delaying abscission.

Studies on changes in enzyme activity in abscission zones have primarily focused on cell wall-degrading enzymes and their genes [2, 3]. The first enzyme proposed to contribute to wall loosening at the site of abscission was endo-β-1,4-glucanase (EGase), or cellulase [4]. Expression of an EGase gene in *Phaseolus vulgaris* increased during ethylene-promoted abscission, was repressed by the application of auxin, and was restricted to the abscission zone tissue [5]. An increase in expression of EGase genes has also accompanied abscission of *Sambucus nigra* leaflets [6], tomato (*Solanum lycopersicum*) flowers [7] and pepper (*Capsicum annuum*) flowers and leaves [8]. At the enzyme level, an increase in EGase activity was also found during leaf, flower, and fruit abscission [9–11]. As the primary site of abscission-related wall breakdown is the pectin-rich middle lamella, research has also focused on pectinolytic enzymes. Increases in the activity of polygalacturonase (PG) have been reported in abscission zones of tomato leaves, flowers and fruit [12, 13], peach (*Prunus persica*) leaves and fruit [10] and leaflets of *Sambucus nigra* [14]. In general, increased PG activity coincides with the loss of tensile strength of the zone and is restricted to the sites where cell separation takes place. Over-expression of the apple (*Malus x domestica*) fruit-specific PG gene *MdPG1* using the constitutive CaMV 35S promoter reduced cell adhesion in leaf abscission zones and induced premature leaf shedding in transgenic apple plants [15]. During pod dehiscence, several PG genes required for cell separation have been identified in *Arabidopsis* [16]. Expansins, extensins and xyloglucan transglycosylase/hydrolases (XTHs) have also been suggested to contribute to cell wall loosening during abscission [3].

Fruit peeling is another process where an abscission zone develops. Here a biologically programmed zone of separation below the peel is responsible for the separation from the flesh. However, unlike in abscission of leaves or flowers, force needs to be applied to separate peel and flesh. The ease and cleanliness of peel separation is a function of cell wall changes in the peel and flesh during ripening, and so the focus of peelability research has been on changes in gene expression and enzyme activity in tissues where detachability develops. Peelability has been most extensively studied in *Citrus* spp. and banana (*Musa* spp.).

In the final stages of rind development in orange (*Citrus sinensis*) there are changes in the cellular structure of the albedo tissue that abuts the juice sacs. The cells enlarge and develop elongated protuberances, resulting in a spongy network of starfish or spider-shaped cells with large intercellular spaces [17]. In easy-peel satsuma mandarins (*Citrus unshiu*), structural changes that happen early in development and changes in pectins result in a loss of adhesion of the albedo to the juice sacs [18]. Changes in mRNA levels of XTH, expansin, extensin, glycine-rich protein and pectin acetylesterase genes were found which may contribute to rind development and the formation of large intercellular spaces in the albedo. Differences in gene expression patterns of expansin, glycine-rich protein and pectin acetylesterase between the albedo and flavedo have been implicated in the tissue separation process [19].

In banana (*Musa acuminata*), the separation of the peel from the pulp occurs along the loculus, the inner face of the skin where the vascular bundles are located. As in abscission zones of leaves or flowers, the transition region between the peel and the pulp is already well distinguished in young fruitlets, having rows of small isodiametrical cells with large, rectangular-shaped airspaces in between the rows [20]. Differential cell wall changes and activity of cell wall enzymes between peel and pulp tissues seem to lead to tissue separation, with softening occurring much faster in the pulp than in the peel. Pectin solubilisation from the cell wall occurred to a greater extent in the pulp than in the peel. PG activity was higher in the peel tissue than in the pulp, suggesting this enzyme played a major role in the softening of banana peel [21, 22]. Two ripening-related pectate lyase genes and *MaXET1*, a fruit-specific xyloglucan transglycosylase (XET) gene, were differentially expressed in the peel and the pulp during ripening [23, 24]. Two extensin gene sequences present in the pulp and the peel were not only distinct from each other but also differentially expressed. In pulp, extensin mRNA was down-regulated during ripening, whereas in peel it was up-regulated [25, 26], perhaps reflecting biochemical events designed to change the structure of the cell wall. Similarly in *Nicotiana plumbaginifolia* stems and roots, up-regulation of extensin RNA has been shown to increase in cells that require reinforcement of their walls [27].

The fruit of one kiwifruit (*Actinidia*) species, *A. eriantha*, typically become peelable when they are ripe and soft [28]. However, there are wide variations in location of peel detachment in the outer pericarp cell layers, and how cleanly the cells debond in this detachment zone. Only two studies have investigated the development of peelability in *Actinidia* species. An initial study compared a hybrid of *A. chinensis* var. *deliciosa* and *A. eriantha* showing good peel detachment with the *A. chinensis* var. *deliciosa* cultivar ‘Hayward’ showing poor peel detachment that is typical for this species [29]. The structure of the fruit skin (dead cell layers and collenchyma-like hypodermis) and outer pericarp layers was quite similar between these two lines, and no structural differences were observed during development of detachment [29]. The mechanical properties of the skin of *A. chinensis* and several *A. eriantha* genotypes have been investigated in a second study that provided information on skin–flesh adhesion, skin compliance in tension, and skin tearing. Substantial differences in peel detachability and peel strength were observed amongst the tested genotypes [30]. In this study, two closely related *A. eriantha* genotypes were investigated; a ‘good-peeling’ (GP) and a ‘poor-peeling’ (PP) genotype (named G2 and G1 in Harker et al., [30]). These two genotypes have been shown to differ substantially in their peeling behaviour - less force was needed to detach the peel of the GP genotype from the flesh, and the peel had higher elastic and plastic components [30].

The aim of the research described in this paper was to determine differences between the GP and PP genotypes with respect to pectin localisation, cell wall monosaccharide composition, gene expression and enzyme activity in cell walls from tissue on both sides of the detachment zone, peel and outer pericarp. Three time points covering a very narrow firmness range associated with development of detachability and changes in skin strength and flexibility were selected to minimise the occurrence of ripening-related softening changes and maximise the likelihood of detecting immunological, molecular and biochemical differences related to peel detachability or required for modification of peel strength and flexibility. Immunolocalisation using monoclonal antibodies directed against pectin epitopes was carried out to give information on pectin localisation and epitope changes during development of the detachment zone, in the peel and the outer pericarp as well as in the zone of detachment itself. Determination of cell wall monosaccharide composition and degree of esterification of cell wall material gave information on differential changes in chemical composition of cell walls of both genotypes over the course of development of the detachment zone. The expression of genes involved in cell wall breakdown and cell wall loosening (e.g. PG, XTH, expansin, pectate lyase) were tested and related to their participation in development of detachabilty and fruit softening. Finally, by comparing pectin-modifying enzyme activities (PG, β-galactosidase) in the peel and the outer pericarp, we sought to evaluate their influence on cell adhesion, and by comparing hemicellulose-modifying transglycosylase and hydrolase activities (XET and xyloglucanase, mannan transglycosylase and endo-β-mannanase, and xylan transglycosylase and xylanase) we sought to understand their effect on cell wall and tissue strengths within those tissue zones.

## Results

### Structural comparisons of two *A. eriantha* genotypes showed the absence of an abscission-type detachment zone

**Fig. 1:**
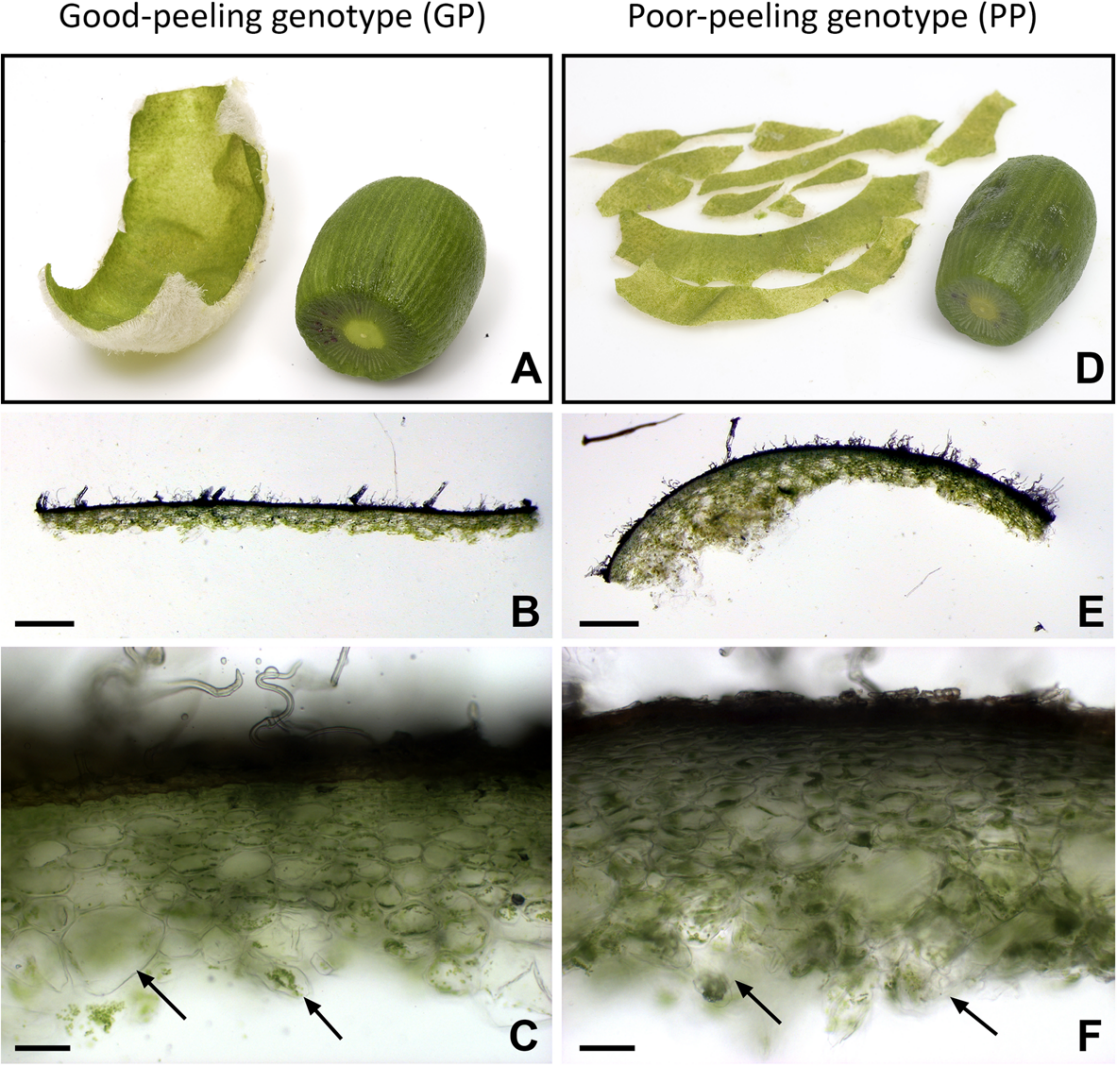
Peeled *Actinidia eriantha* fruit images (**a**, **b**, **d**, **e**) and rough hand sections (**c, f**) of detached peel. Peeled fruit of the good peeling (GP) genotype (**a**) and poor peeling (PP) genotype (**d**), highlighting differences in strength and tearability of the peel. The surface of the fruit after peel detachment is dry in the GP genotype (**a**) and moist in the PP genotype (**d**). The peel of the GP genotype comes off thinly and in even thickness (**b**). The peel of the PP genotype is uneven and thicker (**e**). Most cells in the GP genotype detach cleanly, with complete separation of cell walls in the detachment zone (arrows) (**c**), whereas in the PP genotype, a greater degree of cell rupture is found (arrows) (**f**). Bar A, B = 1 mm; Bar C, D = 100 μm

In ripe fruit of the GP genotype, peel tissue detached in one piece, cleanly and in even thickness from the flesh, indicating strong adherence of cells within the peel tissue (Fig. 1a, b). In the PP genotype, the peel broke often while detaching, leaving behind a moist surface and fruit flesh clumps attached to both peel and outer pericarp. The peel was thicker and uneven compared to the GP genotype (Fig. 1d, e). Hand sections of the peel of ripe fruit showed that most cells in the GP genotype appeared to detach from each other cleanly, showing a complete separation of cell walls in the detachment zone (Fig. 1c). A greater degree of cell rupture and breakage was found in the PP genotype (Fig. 1f).

**Fig. 2:**
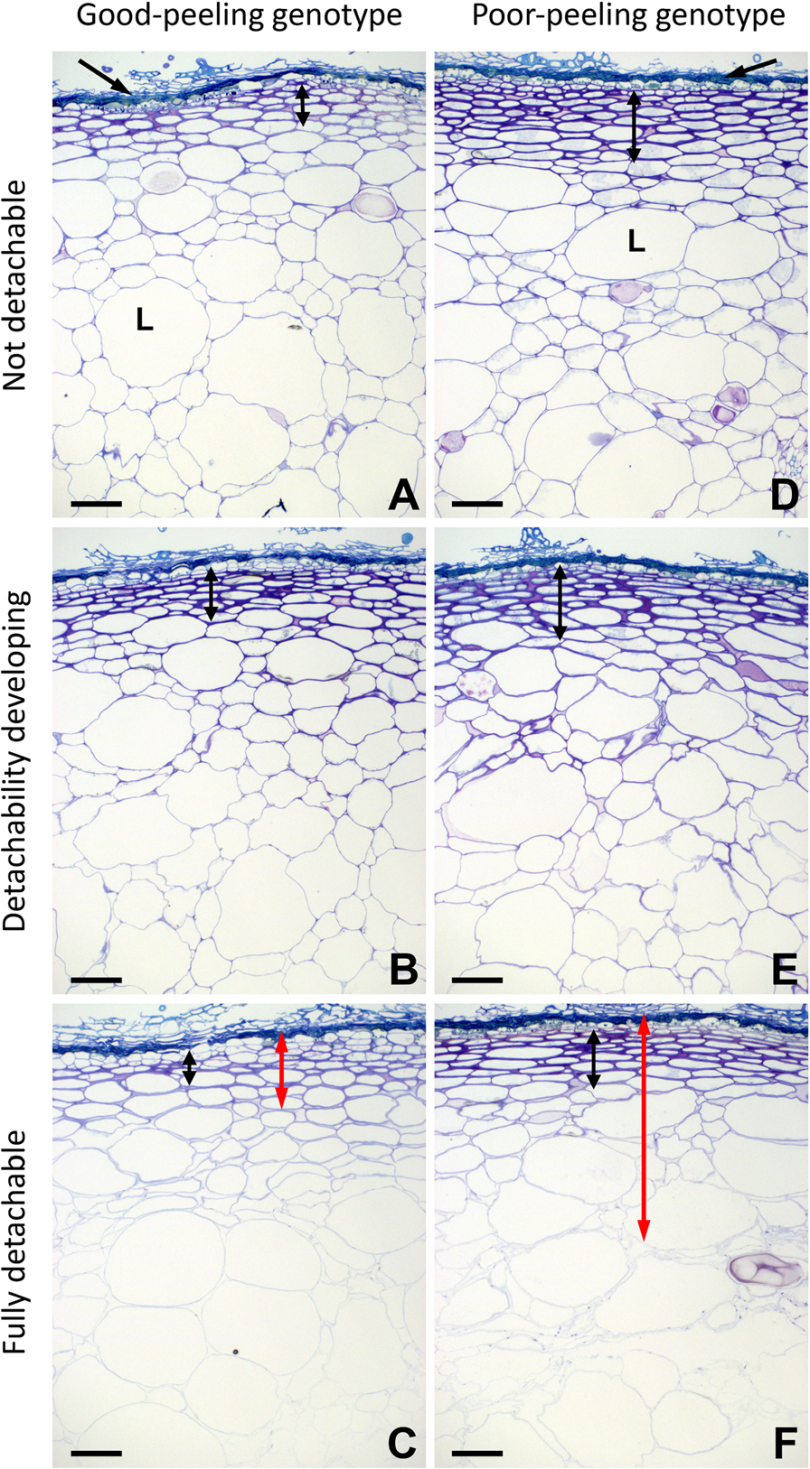
Toluidine blue O-stained sections of skin and outer pericarp tissue from the fruit of the good-peeling (**a**-**c**) and poor-peeling (**d**-**f**) *Actinidia eriantha* genotypes. The surface layers of dead collapsed skin are arrowed in **a**, **d**. Peelability stages: detachability not developed (**a**, **d**); detachability developing (**b**, **e**); fully detachable peel (**c**, **f**). The extent of hypodermal tissue in each genotype is indicated with black double headed arrows (**a**, **d**), and the width of peel detached during peeling with red double headed arrows (**c**, **f**). L, large cell. Bar = 100 μm

The structure of the skin and outer pericarp of fruit from the two *A. eriantha* genotypes was then investigated by light microscopy of Toluidine blue O-stained sections (Fig. 2). In both genotypes the fruit surface consisted of several layers of vertically compressed dead cells with thickened walls from which large multiseriate hairs extended. The dead cell layer overlaid a collenchyma-like hypodermis of radially flattened tightly packed cells with thickened walls (Additional file 1: Figure S1). The hypodermis extended six to eight cell layers deep in the GP genotype and 10–12 cell layers deep in the PP genotype (see double-arrows in Fig. 2a, d). Below the hypodermis is the outer pericarp which consisted of small and large parenchyma cells with thinner cell walls than the hypodermis. In the GP genotype there was a gradual progressive increase in the size of parenchyma cells deeper into the fruit, and large cells were found at a depth of 400–500 *μ*m (Fig. 2a). In contrast, the interface between the hypodermis and thinner parenchyma cells of outer pericarp in the PP genotype was relatively abrupt, and large cells were found adjacent to the hypodermis (Fig. 2d). For comparison, the small parenchyma cells had a maximum cross-sectional dimensions of ~100 μm, and the large cells ~400 μm in both genotypes.

In both genotypes, as the fruit ripened and softened and as detachability developed, changes occurred in the staining of cell walls but no structural differences were observed between the two (Fig. 2b vs c; e vs f). Cells of the outer pericarp showed a decrease in Toluidine blue O staining, indicating loss of cell wall integrity, as well as swelling and cell separation (Fig. 2c, f), changes typical for kiwifruit softening [31]. Stain intensity was retained to a greater extent in cells close to the hypodermis, and also in large cells in the outer pericarp compared with small cells. In the PP genotype, cells of the hypodermis showed little change in stain intensity (Fig. 2d–f), whereas staining of hypodermal cells in the GP genotype seemed to reduce as detachability developed (Fig. 2a–c). No morphologically identifiable abscission zone was apparent at any developmental stage investigated.

### Changes in pectin methylesterification and galactan side chain distribution are associated with the development of detachment

Immunolocalisation was performed using three antibodies with specificity for different epitopes in pectin. JIM5 recognises unesterified or partially methyl-esterified epitopes on the homogalacturonan (HG) backbone of pectin; JIM7 recognises partially methyl-esterified HG epitopes (but not unesterified regions) [32]; whilst LM5 recognises linear galactan tetrasaccharide epitopes in (1–4)-β-D-galactan side chains of rhamnogalacturonan-I pectin [33].

**Fig. 3:**
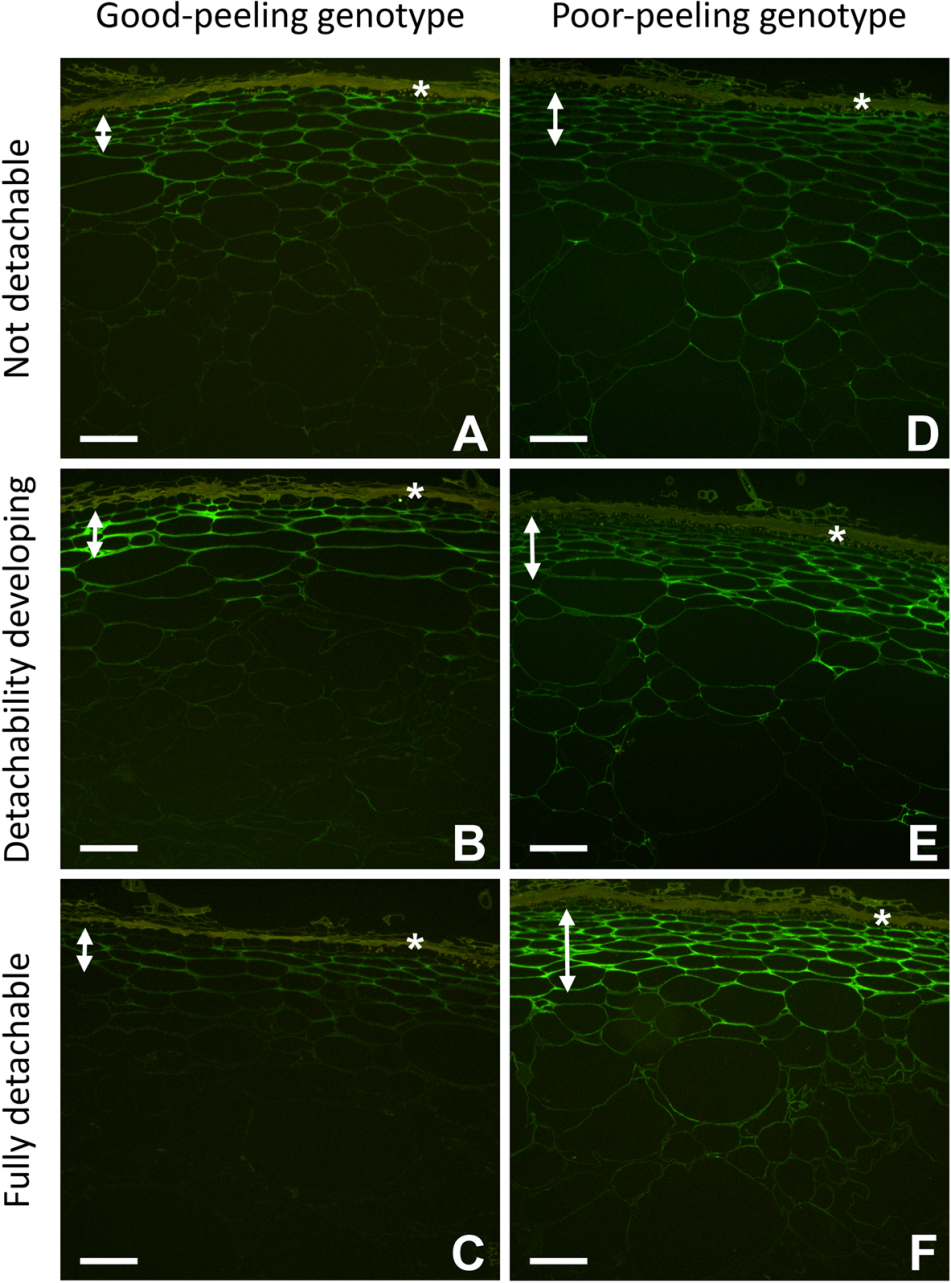
Immunolabelling of good-peeling (**a**-**c**) and poor-peeling (**d**-**f**) genotypes of *Actinidia eriantha* fruit using the JIM5 antibody conjugated to Alexa™488 (green) for detection of unesterified and low-esterified homogalacturonan epitopes in the pectin backbone. Position of the auto-fluorescing dead skin layer indicated by (*), and double headed arrows indicate the extent of hypodermal tissue in each genotype. Peelability stages: detachability not developed (**a**, **d**); detachability developing (**b**, **e**); fully detachable peel (**c**, **f**). Bar = 100 μm

JIM5 labelling was not particularly strong in fruit of both genotypes before development of detachability (Fig. 3a, d). In the GP genotype, labelling intensity seemed to decrease just beneath the hypodermal layers, whereas the hypodermal and outer pericarp areas were labelled with similar intensity in the PP genotype. The cell wall labelling pattern was similar in both genotypes, with the tricellular junctions being most intensely labelled (Fig. 3a, d). In fruit where detachability had started to develop, labelling increased in both genotypes, but the overall pattern of labelling remained the same. Labelling in the PP genotype extended well into the outer pericarp whilst in the GP genotype it did not (Fig. 3b, e). In fruit where detachability was fully developed (Fig. 3c, f), labelling had become weaker in the GP genotype and was absent from the outer pericarp (Fig. 3c). In contrast, in the PP genotype labelling was strong in the hypodermal region and immediately adjacent outer pericarp and was weakly retained in other outer pericarp regions. Overall, in the PP genotype, labelling in the hypodermal region and 2–3 cell layers of outer pericarp seemed to increase over development of detachability, whereas the remaining outer pericarp labelled more weakly but similarly to earlier stages (Fig. 3f).

**Fig. 4:**
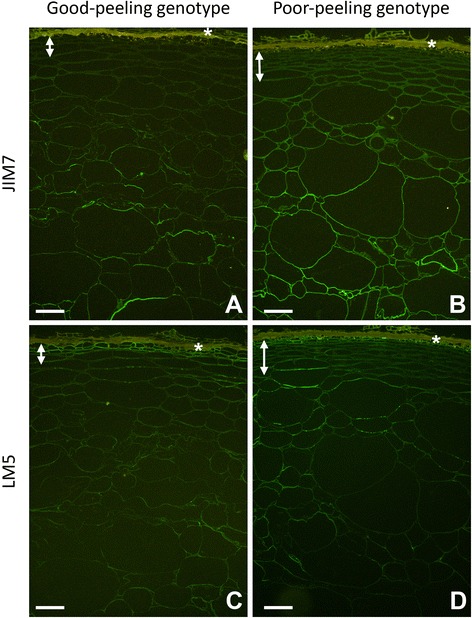
Immunolabelling of good-peeling (**a**, **c**) and poor-peeling (**b**, **d**) genotypes of *Actinidia eriantha* fruit when detachability was developing (DD) using JIM7 for detection of partially methyl-esterified homogalacturonan epitopes in the pectin backbone (**a**, **b**) and LM5 for detection of (1–4)-β-D-galactan side chains of rhamnogalacturonan-I pectin (**c**, **d**). Both antibodies were conjugated to Alexa™488 (green). Position of the auto-fluorescing dead skin layer indicated by (*), and double headed arrows indicate the extent of hypodermal tissue in each genotype. Bar = 100 μm

Immunolabelling with JIM7 and LM5 was generally less intense than with JIM5 and declined during softening, although the overall pattern of labelling of each antibody was retained to the stage when detachability had started to develop (Fig. 4). At this stage labelling with JIM7 was quite weak in the GP genotype, with the hypodermal region and immediately adjacent outer pericarp cell layers showing little or no label. Stronger labelling occurred deeper in the outer pericarp, although only stretches of cell walls were labelled, the most intense being associated with large cells (Fig. 4a). The PP genotype showed weak but consistent labelling throughout the hypodermal and adjacent outer pericarp cell walls. Labelling increased in intensity deeper into the outer pericarp, where it was much more uniform than in the GP genotype (Fig. 4b). Immunolabelling with JIM7 and LM5 at the non-detachable stage is shown in Additional file 2: Figure S2.

Labelling with LM5 in the GP genotype was mostly restricted to the three to four hypodermal cell layers below the skin, with only weak labelling in the outer pericarp (Fig. 4c). In the PP genotype, again hypodermal cells immediately below the skin were labelled, with the rest of the hypodermis having only weak labelling. Stronger, but discontinuous, labelling was found in the outer pericarp (Fig. 4d).

### Differences in peel detachability are associated with changes in cell wall monosaccharide composition

The immunolocalisation results suggest that changes in the localisation and composition of cell wall pectin are involved in the development of detachability in *A. eriantha*. Investigating the monosaccharide composition of cell walls of both genotypes (Table 1), the total non-cellulosic monosaccharide content of cell wall material significantly decreased in the peel tissue of the PP genotype during development of detachability, whereas in the GP genotype and the outer pericarp of the PP genotype it remained relatively constant. The galactosyl content in the cell walls showed little change over development of detachability; however, the cell walls of the PP genotype had overall more galactosyl residues in both peel and outer pericarp than that in the GP genotype. Cell wall galacturonyl acid-, rhamnosyl-, and arabinyl residues (pectin-related) and fucosyl-, xylonyl-, and mannonyl residues (hemicellulose-related) were similar between the two genotypes and showed little change while detachability developed, except for the glucosyl content, which decreased in cell walls of both genotypes.

**Table 1:** Non-cellulosic monosaccharide composition and degree of methylesterification (DE) of cell wall material prepared from peel and outer pericarp of the good-peeling and the poor-peeling *Actinidia eriantha* genotypes over the development of detachment

	Good-peeling genotype			Poor-peeling genotype		
MS	ND	DD	FD	ND	DD	FD
Peel						
Rha	5.9 ± 0.8	6.2 ± 0.7	5.1 ± 0.3	4.9 ± 1.0	5.8 ± 1.6	5.2 ± 1.1
Ara	20.6 ± 3.6	21.7 ± 4.7	17.0 ± 2.3	16.3 ± 4.2	17.9 ± 5.2	18.0 ± 6.0
Gal	29.3 ± 4.7	31.1 ± 5.8	23.3 ± 2.8	39.3 ± 8.9	39.7 ± 11.0	40.5 ± 11.0
UA	237.3 ± 31.7	234.6 ± 27.3	211.7 ± 26.6	240.4 ± 41.5	248.4 ± 36.0	202.8 ± 31.9
Fuc	1.1 ± 0.5	0.9 ± 0.3	0.8 ± 0.1	1.4 ± 0.4	1.9 ± 0.9	1.6 ± 0.7
Xyl	25.5 ± 9.2	22.8 ± 6.0	19.7 ± 1.7	20.9 ± 5.2	26.7 ± 10.3	24.9 ± 9.8
Man	7.0 ± 0.8	6.8 ± 0.7	5.4 ± 0.5	7.4 ± 1.3	8.1 ± 2.1	7.7 ± 1.9
Glc	21.1 ± 2.9	18.6 ± 2.1	12.6 ± 0.3	34.7 ± 5.5	26.3 ± 3.1	20.0 ± 2.3
TS	294.8 ± 20.7	341.0 ± 20.1	297.2 ± 7.2	388.0 ± 26.2	353.2 ± 30.8^a^	291.6 ± 32.3^b^
DE	57.8 ± 2.7	62.1 ± 4.3	62.7 ± 8.1	55.5 ± 4.2	53.6 ± 5.9	66.7 ± 4.9^b^
Outer pericarp						
Rha	4.6 ± 2.2	5.1 ± 0.2	4.5 ± 0.6	5.9 ± 1.7	3.9 ± 1.1	3.8 ± 1.3
Ara	14.3 ± 2.5	14.5 ± 4.5	15.2 ± 6.0	18.9 ± 4.3	14.1 ± 4.3	14.0 ± 3.4
Gal	19.0 ± 3.5	19.7 ± 4.0	16.9 ± 4.3	30.5 ± 3.5	24.3 ± 4.8	23.5 ± 2.3
UA	249.2 ± 57.2	246.9 ± 32.1	264.2 ± 62.2	256.4 ± 39.8	306.4 ± 55.1	318.7 ± 30.9
Fuc	2.4 ± 0.6	2.6 ± 0.5	2.7 ± 0.6	5.9 ± 1.3	4.3 ± 1.1	4.5 ± 0.6
Xyl	16.6 ± 3.3	18.3 ± 5.5	16.3 ± 5.4	25.9 ± 5.5	17.8 ± 4.6	18.5 ± 3.5
Man	5.8 ± 1.3	5.8 ± 0.3	5.1 ± 0.3	10.5 ± 2.6	7.9 ± 1.4	7.7 ± 1.0
Glc	25.6 ± 15.6	25.1 ± 7.7	8.3 ± 1.1	29.0 ± 13.5	19.5 ± 7.9	12.5 ± 4.5
TS	334.7 ± 28.2	334.1 ± 28.4	336.7 ± 16.5	375.0 ± 25.7	390.4 ± 13.3	398.4 ± 8.3
DE	69.2 ± 5.6	72.4 ± 9.2^^^	72.2 ± 5.2^^^^	60.1 ± 6.4	52.6 ± 4.1	56.1 ± 4.1

The monosaccharide composition is expressed in μg anhydro monosaccharide per mg cell wall material, and DE as the molar ratio of methanol to uronic acid in %. Peelability stages: ND, detachability not developed; DD detachability developing; FD, fully detachable peel. TS, total non-cellulosic sugars (TFA-hydrolysable neutral monosaccharides plus uronic acid). Rha, rhamnose; ara, arabinose; gal, galactose; UA, uronic acid; fuc, fucose; xyl, xylose; man, mannose; glc, glucose. Data are means of *n* = 4 ± standard deviation. (^) and (^^) represent statistical significance (*p* value <0.05) between GP and PP at the same developmental stage (DD or FD)

^a^represents statistical difference of DD compared to FD

^b^between NP and FD within the PP genotype

In the cell wall material of the peel tissue, the degree of esterification (DE) was between 55 and 65% in both genotypes; however, it increased in the PP genotype while detachability developed. At the fully detachable stage (FD), the DE was significantly higher than at the non-detachable stage (ND) in the PP genotype. In the outer pericarp, the DE was higher in the GP genotype (~70%) than in the PP genotype (between 50 and 60%), and significantly so at the ‘detachability developing’ (DD) and ‘fully detachable’ (FD) stage compared with the DE in the PP genotype (Table 1).

### Gene expression changes during the of development of detachability

**Fig. 5:**
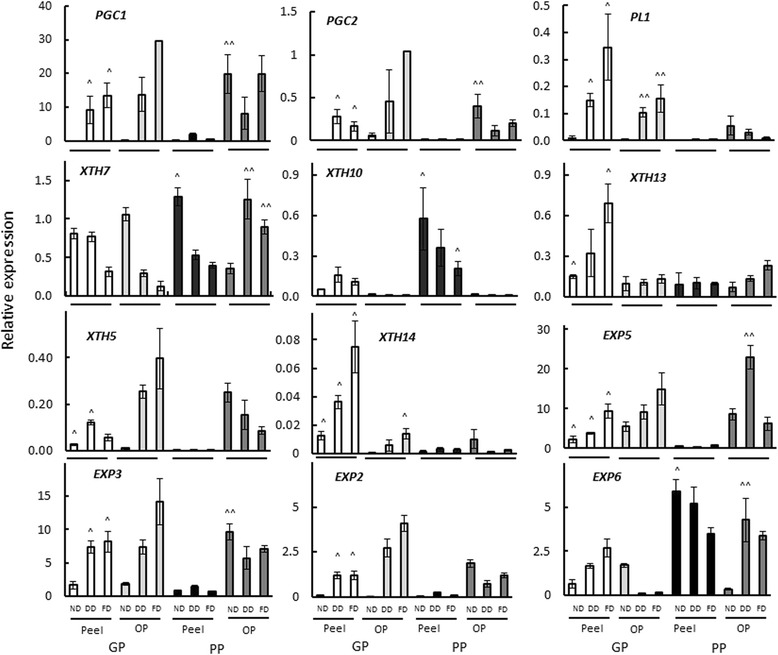
Expression of polygalacturonase (PG), pectate lyase (PL), xyloglucan transglycosylase/hydrolase (XTH) and expansin (EXP) genes in *Actinidia eriantha* good-peeling (GP; white and light grey bars) and poor-peeling (PP; black and dark grey bars) genotypes over the development of detachability. Note different scales in panels. OP = outer pericarp. Peelability stages: ND, detachability not developed; DD, detachability developing; FD, fully detachable peel. *n* = 3 ± standard error. (^) represents statistical significance between peel tissue and (^^) between outer pericarp tissue of GP and PP at the same developmental stage (ND, DD, or FD) (*p* value <0.05)

Reverse transcriptase quantitative PCR (RT-qPCR) using gene specific primers (Additional file 3: Table S1) was used to examine differences in the expression of genes associated with pectin and hemicellulose modification during the development of detachability. Expression of PG genes *PGC1* and *PGC2* was detected in the peel and outer pericarp of the GP and the PP genotypes (Fig. 5). The peel of the GP genotype showed significantly higher expression of both PG genes when detachability developed and at the fully detachable stage when compared to the corresponding stages of the peel of the PP genotype. A pectate lyase gene (*PL1*) described in Atkinson et al., 2011 [34] was also expressed in both genotypes, albeit at a lower level in the PP genotype (Fig. 5).

Fourteen XTH genes have previously been shown to be expressed in kiwifruit [35]. Five of these genes, *XTH5*, *7*, *10*, *13* and *14,* were expressed at moderate to high levels in both *A. eriantha* genotypes during development of detachability (Fig. 5). *XTH7* was highly expressed in the peel and outer pericarp of both genotypes. Whereas *XTH10* was significantly higher expressed in the peel of the PP genotype, the expression of *XTH5, 13* and *14* was significantly higher in the peel tissue of the GP genotype. *XTH1* and *XTH6* were only expressed at low level, whilst *XTH2–4, 8, 9, 11* and *12* showed no expression at all.

Based on expressed sequence tag (EST) expression data from kiwifruit [36], eight expansin genes (*EXP1–8*) were examined in this study (GenBank KY496691–98, see Additional file 3: Table S1). Four genes (*EXP2, 3, 5* and *6*) were shown to be highly expressed over the development of detachability. *EXP1*, *4* and *7* were expressed at low to medium levels (Additional file 4: Figure S3), whilst *EXP8* expression was not detected. In the peel, *EXP2, EXP3* and *EXP5* showed significantly higher expression in the GP genotype especially when detachability was developing and at the fully detachable stage, whereas *EXP6* showed significantly higher expression in the PP genotype at all developmental stages. Expression of these four genes significantly increased in the peel of the GP genotype, whereas *EXP6* decreased in the PP genotype over development of detachment. *EXP2*, *3* and *5* were expressed in the outer pericarp of both genotypes; *EXP6* was highly expressed in the PP genotype but not in the GP genotype (Fig. 5).

### Differences in the abundance and activity of pectinolytic enzymes and expansin during the of development of detachability

**Fig. 6:**
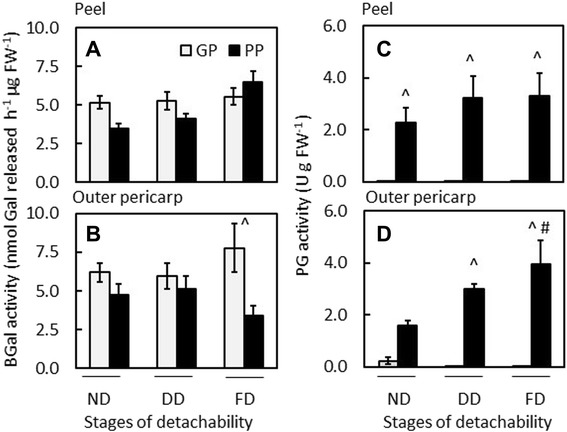
Activity of the pectinolytic enzymes β-galactosidase (BGal) (**a**, **b**); and polygalacturonase (PG) (**c**, **d**) in low salt extracts of the good-peeling (GP; white bars) and poor-peeling (PP; black bars) *Actinidia eriantha* genotypes over development of peelability. Peelability stages: ND, detachability not developed; DD, detachability developing; FD, fully detachable peel. BGal, *n* = 4 ± standard error; PG, *n* = 3 ± standard error. (^) represents statistical significance between GP and PP at the same developmental stage (ND, DD, or FD). In D, (#) represents statistical significance of FD compared to ND within the PP genotype

The activities of two pectinolytic enzymes, PG and β-galactosidase (BGal), were monitored during the development of detachability. BGal activity was similar between the two genotypes (Fig. 6a, b) except in the outer pericarp at the fully detachable (FD) stage, where BGal activity was significantly higher in the GP genotype than in the PP genotype (Fig. 6b). Most BGal activity was extracted in a low salt (LS) buffer (80–90%), suggesting that the enzyme activity was freely soluble and not cell wall-bound. Western blotting using an apple BGal antibody indicated the presence of several isoforms in both genotypes, with the peel containing more bands than the outer pericarp (Additional file 5: Figure S4A, B).

Polygalacturonase activity was overall significantly higher in the PP genotype than in the GP genotype in both tissues throughout development of detachment (Fig. 6c, d). In the GP genotype, only a small amount of PG activity was detected in the outer pericarp before detachment started to develop (DD) (Fig. 6d), but activity was undetectable at all other stages and in the peel (Fig. 6c, d). In the PP genotype, PG activity increased over development of detachability and was significantly higher at the fully detachable stage (FD) compared with the non-detachable stage (ND) in both tissues (Fig. 6c, d). Polygalacturonase activity was detected only in LS extracts, indicating that this kiwifruit PG was freely soluble and not cell wall-bound.

Some of the activity accredited to PG in the gel diffusion assay may have derived from a pectate lyase enzyme acting on the HG backbone of the pectin substrate, as a pectate lyase gene was expressed in both genotypes (Fig. 5). However, western analysis with an antibody directed against kiwifruit pectate lyase did not reveal the presence of protein in either genotype despite gene expression.

Western analyses using an antibody raised against kiwifruit EXP3 protein showed at least two expansin protein isoforms were present in both genotypes in the outer pericarp and peel tissue. A third isoform, with lower molecular weight, was present in the peel of the GP genotype (Additional file 5: Figure S4C, D).

### Changes in hemicellulose-modifying enzyme activities during the development of detachability

Xyloglucan transglycosylase/hydrolase (XTH), mannan transglycosylase/hydrolase and xylan transglycosylase/hydrolase are enzymes with dual activity; they can either remodel their respective hemicellulose substrates by transglycosylation (XET, mannan transglycosylase, xylan transglycosylase), or hydrolyse them (xyloglucanase, endo-β-mannanase, xylanase).

**Fig. 7:**
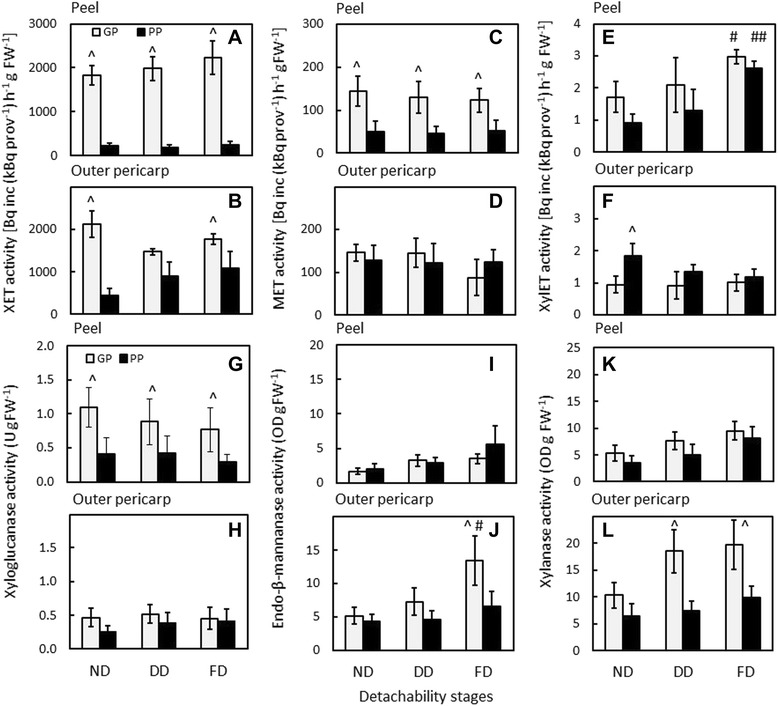
Activity of transglycosylases and hydrolases acting on hemicelluloses in the peel and outer pericarp of the good-peeling (GP; white bars) and poor-peeling (PP; black bars) *Actinidia eriantha* genotypes. Activities: xyloglucan transglycosylase (HS extract) (**a**, **b**); mannan transglycosylase (MET; HS extract) (**c**, **d**); xylan transglycosylase (XylET; HS extract) (**e**, **f**); xyloglucanase (LS extract) (**g**, **h**); endo-β-mannanase (LS extract) (**i**, **j**); xylanase activity (HS extract) (**k**, **l**). LS, low salt; HS, high salt. Peelability stages: ND, detachability not developed; DD detachability developing; FD, fully detachable peel. *n* = 3 ± standard deviation. (^) represents statistical significance between GP and PP at the same developmental stage (ND, DD, or FD). In E and J, (#, ##) represent statistical significance of FD compared to ND within a genotype (# GP, ## PP)

The GP genotype had high levels of XET activity in the peel and the outer pericarp throughout the development of detachability. The PP genotype had only low levels of XET activity in the peel tissue throughout development of detachability, whereas in the outer pericarp, XET activity increased while detachability developed. However, XET activity in the outer pericarp was consistently lower than in the GP genotype, and significantly so when detachability had not developed yet (ND stage) and when peel was fully detachable (FD stage) (Fig. 7a, b). The bulk of XET activity was released in high salt (HS) buffer, showing that XET was mostly cell wall bound.

Mannan transglycosylase activity was extracted in LS and in HS buffer, indicating freely soluble and cell wall-bound isoforms. The same activity trends were observed in both LS and HS extracts. Figures 7c and d show the results for mannan transglycosylase in the HS extract. Whereas enzyme activity was similar in both genotypes in the outer pericarp over development of detachability, it was significantly higher in the GP genotype in the peel tissue at all developmental stages.

Xylan transglycosylase activity was on average higher in the peel of the GP genotype than in the PP genotype before detachability developed (ND stage) and when detachability was developing (DD stage). In both genotypes, xylan transglycosylase activity in the peel increased over development of detachability, and was for both genotypes significantly higher in the fully detachable stage than in the ND stage (Fig. 7e). In the outer pericarp, xylan transglycosylase activity was similar in both genotypes when detachability developed (DD) and at the fully detachable stage (FD). At the non-detachable stage (ND), xylan transglycosylase activity was significantly higher in the PP genotype compared to the GP genotype (Fig. 7f).

Xyloglucanase activity was mainly extracted in LS buffer. Enzyme activity was significantly higher in the peel of the GP genotype than in the PP genotype at all developmental stages, and decreased slightly in the GP genotype over the course of development of detachability (Fig. 7g). The peel tissue of the PP genotype and outer pericarp from both genotypes showed only low levels of activity at all developmental stages (Fig. 7h).

Endo-β-mannanase activity was mainly extracted in LS buffer. The activity was low in peel tissue, and increased slightly over development of detachability in both genotypes (Fig. 7i). In the outer pericarp, endo-β-mannanase activity increased in the GP genotype over development of detachability; at the fully detachable stage (FD), the activity was significantly higher than at the not detachable (ND) stage of this genotype. At the fully detachable stage, endo-β-mannanase activity in the outer pericarp was significantly higher in the GP genotype than in the PP genotype (Fig. 7j).

Xylanase activity was extracted in HS buffer. In the peel tissue, xylanase activity increased slightly over development of detachability and was similar in both genotypes at all developmental stages (Fig. 7k). In the outer pericarp, xylanase activity increased in the GP genotype compared with the PP genotype over the course of development of detachability; the activity was significantly higher in the GP genotype when detachment developed (DD stage) and when peel was fully detachable (FD stage) compared with the PP genotype (Fig. 7l).

Endo-β-1,4-glucanase activity against various soluble cellulose substrates was not detected in either genotype (see Method section).

Western analyses with an antibody raised against kiwifruit XTH7 showed multiple bands in peel and outer pericarp of both genotypes (Additional file 5: Figure S4E, F), indicating that the antibody cross-reacted with other XTH proteins. Band intensity was much stronger in the GP genotype than in the PP genotype. Western analysis with antibodies directed against XTH5 from kiwifruit, papaya endoxylanase, and endo-β-mannanase from tomato seeds did not reveal the presence of XTH5, xylanase or endo-β-mannanase protein in either genotype.

## Discussion

Abscission and dehiscence zones, as well as peel detachment areas in the fruits of banana or mandarin, have morphologically distinct structural features that predict where cell separation will take place. In fruit of *A. eriantha,* however, neither skin nor outer pericarp show structural features that would predict either where cell detachment will occur, or how clean the separation will be. Although the hypodermis in both genotypes showed considerable structural difference from the outer pericarp tissue, the adhesion of hypodermal cell layers (plus a few underlying outer pericarp cell layers) remained relatively strong throughout ripening. Instead, detachment occurred in the outer pericarp region further below, once a peeling force had been applied. Our research showed that the main aspects that seem important for development of peelability are differences in degree of methylesterification, galactose loss, and PG and XTH enzyme action, leading to spatially distinct softening zones (peel and underlying flesh) that are detachable once the fruit is soft.

The differential methylesterification pattern of the pectic HG backbone as observed by immunolocalisation made the location where detachment occurred apparent. In the GP genotype, JIM5 labelling of low-esterified HG ended sharply just below the hypodermis in the outer pericarp region where the detachment took place. JIM7 labelling of highly-esterified HG started in the same region and extended further into the outer pericarp, whilst the hypodermis was hardly labelled at all. In the PP genotype, labelling with both antibodies showed gradual changes and extended from the hypodermis deep into the outer pericarp. These results suggest that, in the GP genotype, the cells at the interface between JIM5 and JIM7 labelling provide a discontinuity in strength where the peel will detach, whereas in the PP genotype, no such zone exists. HG is the major component of middle lamellae and hence strongly influences the strength of cell adhesion in a tissue. It is deposited in cell walls in a highly methyl-esterified form but is then de-esterified [1, 37]. Domains of adjacent de-esterified galacturonic acid residues can be cross-linked by calcium, resulting in gel formation that contributes to intercellular adhesion and also conveys different mechanical properties to the tissue. In vitro analysis of model pectin gels indicated that the compressive strength, elasticity, and water-holding capacity as well as the porosity of gels was significantly influenced by both the DE and the esterification pattern of HG domains [37]. The chemically determined DE of HG in *A. eriantha* cell walls was over 50% in the peel and outer pericarp cell walls of both genotypes at all developmental stages, indicating highly-esterified HG chains in both genotypes. However, only the PP genotype labelled with JIM7 specific for high-methylesterified HG, whereas in the GP genotype little to no labelling occurred. This suggests that either the epitope specificity of JIM7 is not met by the esterification pattern present in cell walls extracted from the GP genotype, or that the epitopes are masked in the cell wall and inaccessible for antibody binding.

Galactans have been argued to modify mechanical properties of cell walls. In tomato fruit, galactans are abundant in the pericarp of green, firm tomatoes but absent from the locular gel and the epidermal and subepidermal cells [33]. In pea cotyledons, they appear at a time late in development prior to seed maturation and dehydration. Mechanical compressive testing of cotyledons before and after galactan appearance showed that the walls with galactan were twice as firm as the ones with no detectable galactan [38]. In the GP *A. eriantha* genotype, only the outer hypodermal layers showed high label intensity for galactan; labelling in the PP genotype on the other hand was present throughout the peel and outer pericarp. Additionally, cell wall galactosyl content were significantly higher in the PP genotype. This may point to a greater firmness or stiffness of the peel of the PP genotype, and – together with the much lower transglycosylase activities in this tissue – may lead to easy rupture or tearing of the peel, whereas the peel of the GP genotype is more elastic or flexible [30]. Although BGal activity was high, neither genotype lost great amounts of galactose from the cell wall while detachability developed. As in tomato, isoforms of the whole BGal gene family may be responsible for concerted degradation of galactan observed in kiwifruit. In *A. eriantha*, several isoforms of BGal were present as shown by western analysis and, as in tomato, they may have various roles during fruit growth and ripening. In tomato, only the suppression of a BGal isoform appearing very early in development (isoform TBG6) had a major impact on tomato skin structure, resulting in severe fruit skin cracking [39]; a lower cell wall galactosyl content and fruit firmness compared with those in the wild-type were observed only when the fruit was still developing, but not later when the fruit was ripe.

In *A. eriantha*, cell wall compositional changes during development of detachment were subtle. However, differences in enzyme activity were observed that point to differential softening of peel and outer pericarp tissue as a major factor in development of peelability. The GP genotype showed higher expression of *PGC1* and *PGC2* genes than the PP genotype during development of detachment, but did not show PG activity. To examine whether there were any mutations in the PG gene of the GP genotype, two full-length PG genes were obtained from outer pericarp tissue of both *A. eriantha* genotypes and their sequences compared (see Additional file 3: Table S1 for methodology). The first gene *PGC1* (GenBank KY496689), showed high homology (> 98% amino acid identity) to a partial PG gene previously isolated from ripe *A. chinensis* var. *chinensis* fruit (AF152756) [40]. The second gene, *PGC2* (KY496690), showed ~75% amino acid identity to *PGC1*. Sequencing of these full-length PG sequences from both genotypes showed no frame-shift or other mutations that would lead to a non-functional protein in the GP genotype.

As PG activity was only detected in the PP genotype, enzyme-related HG breakdown in the pectin domain could lead to localised softening and weakening in peel and outer pericarp. This might account for the lower peel elasticity measured in this genotype [30] and the easy tearing and fragmentation of this tissue upon detachment. Gel strength and ultimately cell adhesion is influenced by the overall length of HG. The longer the HG chains, the stronger the gel, either at low DE by calcium cross-linking, or at high DE by hydrophobic interactions [37]. Hence in the GP genotype, any HG gels formed in peel and outer pericarp cell walls might be stronger than in the PP genotype, as HG chains of high molecular weight range would be maintained in this genotype due to lack of PG activity. Differential softening in the absence of an abscission zone is also seen in ‘banana finger drop’, where fruit break off the crown when they are ripe. Banana genotypes showing frequent finger drop had higher levels of water-soluble pectin, lower CDTA-soluble pectin levels and higher PG and pectate lyase activity levels in the pedicel tissue adjacent to the rupture area than genotypes whose fruit rarely break off the crown. Hence it was concluded that breakage occurred due to localised softening and weakening in this tissue area due to significant changes to the cell wall pectin domain mediated by PG, whereas other areas (apart from normal softening changes) remained unaffected [41–43].

In *A. eriantha,* transglycosylase activities were significantly higher in the peel tissue of the GP genotype than in the PP genotype, and two XTH genes were almost exclusively expressed in this tissue. Re-arrangement of hemicelluloses by transglycosylases in peel cell walls of the GP genotype may ensure that the tissue remains strong but also more flexible and elastic when fully peelable, and that the peel might therefore tear less. Correspondingly, the lower transglycosylase activities in the PP genotype may lead to a weaker skin that easily tears. Transglycosylase activities are reported to play a role in the outermost tissue layers of other fruit. Thompson et al., [44] reported the importance of XET in epidermal cell walls of developing tomato, where XET activity was found to be proportional to the relative expansion rate of fruit until growth ceased. Johnston et al., [45] found increased levels of xylan transglycosylase activity in apple peel compared with that in cortex tissue, and Schröder et al., [46] reported highest mannan transglycosylase activity in tomato peel.

Xyloglucan transglycosylase activity was also present in the outer pericarp of both genotypes, and increased over development of detachment in the PP genotype. These activities may reflect changes that are mainly softening related, since an increase in XET activity in the outer pericarp tissue during softening has been found in *A. chinensis* var*. deliciosa* [47] and in two *A. chinensis* var. *chinensis* genotypes [48], kiwifruit species that are not peelable. In *A. eriantha*, this XET activity may be related to *XTH7*, as only this gene is highly expressed in the outer pericarp of both genotypes.

Xyloglucanase, endo-β-mannanase and xylanase activities were significantly higher in the outer pericarp of both genotypes. Endo-β-mannanase and xylanase activities appeared to increase in the outer pericarp as detachment developed and this occurred earlier in the GP genotype than in the PP genotype. The more advanced hydrolysis of these hemicelluloses in the outer pericarp of the GP genotype may aid cell separation and detachment of the peel from outer pericarp. Chanliaud et al., [49] found strong and complementary mechanical effects for transglycosylating and hydrolytic enzymes using cell wall composite material made of cellulose and xyloglucan. In this system, extensive cross-linking of cellulose with xyloglucan resulted in a weaker, less stiff, and more extensible structure than with cellulose alone [50]. Treatment with a xyloglucanase degraded most of the surface cellulose-bound and cross-linking xyloglucans, and the resulting composite material became more stiff and stronger, including a decrease in creep behaviour, thereby providing evidence that hydrolytic enzymes can have a strengthening effect on cell walls. Xyloglucan transglycosylase activity had a complementary effect on the composite, as it increased its ability for molecular rearrangement as measured by creep. It was concluded that xyloglucanase and XET action can lead either to increased strength (hydrolase) or to enhanced viscoelasticity (transglycosylase) of the xyloglucan/cellulose networks *in muro* [49]. In our study, the peel of the GP genotype had higher xyloglucanase and higher XET activity than the PP genotype. One could speculate that the xyloglucanase activity may be strengthening the tissue to prevent breakage during the peeling action, and that re-arrangement of the xyloglucan in the peel tissue of the GP genotype mediated by XET resulted in higher flexibility of the peel tissue. Whether effects like this also play a role with hydrolases and transglycosylases acting on mannans and xylans in the cell wall remains to be determined.

## Conclusions

A novel cell separation mechanism has been found in kiwifruit in the absence of a morphologically identifiable abscission zone, making the fruit peelable. Examining two closely-related kiwifruit genotypes with contrasting detachment behaviour, cell wall features were identified that were important in the development of detachment using immunolocalisation, gene expression analyses and enzyme activity assays. The main differences between genotypes were in degree of methylesterification, galactose loss, and PG and XTH enzyme action. Xyloglucan-, mannan-, and xylan transglycosylase activities were higher in peel tissue than in the underlying flesh, ensuring that the peel tissue remained strong but also more flexible and elastic during detachment. These findings broaden the tasks of these enzyme activities from being involved in cell expansion to being involved in determining the mechanical properties of plant tissues. These results provide direction for future studies aimed at investigating transglycosylase action in other plant surface tissues.

## Methods

### Fruit material

Fruit of the *A. eriantha* Benth. GP and PP genotypes were grown at Plant & Food Research, Te Puke, New Zealand. The two genotypes were selected in 1997 among seedling vines originating from two generations of breeding in New Zealand, involving crosses made in 1991 and 1994. The original parents used for breeding came from three introductions of seed into New Zealand from China: 1976, gifted by Prof. Li Lai-Yung, University of Fujian; 1981 and 1988, gifted by Prof. Liang Chou-fen, Guangxi Institute of Botany, Guilin, China.

One hundred and fifty fruit of each genotype were harvested from about ten-year-old perennial vines at a firmness of ~60 N and ripened at 20 °C without the addition of exogenous ethylene. For firmness measurements, a thin slice of skin was removed from fruit using a scalpel and firmness measured (Instron Model 4301, Canton, MA) by driving a 2.5 mm diameter flat-tipped probe into the flesh at a speed of 4 mm s^−1^. Outer pericarp and peel tissues were sampled at three stages according to the peeling behaviour of the GP genotype: before fruit was peelable (not detachable ‘ND’; 12–16 N); when fruit started to become peelable (detachability developing ‘DD’ and characterised by the peel coming off in patches but tears easily; 9–11 N); when fruit was fully peelable (fully detachable ‘FD’; 4–7 N).

For each developmental stage, tissue of ~30 fruit was collected. Fruit were sampled by removing hair followed by thinly removing peel (~0.4 mm) and then adjacent outer pericarp tissue (~1 mm) with a scalpel. Tissue was cut into small pieces, immediately frozen in liquid N_2_ as bulk samples, and stored at −80 °C. Developmental series were collected from fruit from two seasons; for cell wall analyses fruit material from both seasons was used, and for gene expression and enzyme activity analyses fruit material from only one season was used.

### Light microscopy and immunolabelling

For localisation of cellular detachment, scored strips of skin were removed using an Instron (Model 4301) [30]. Segments of the peel were fixed in 4% formaldehyde in 0.1 M phosphate buffer (pH 7.2), washed in buffer and hand-sectioned in a direction parallel to that of peeling using a pair of safety razor blades separated by two coverslips. Segments were mounted in buffer and observed using a Vanox microscope and a Leica FLZIII stereofluorescence microscope (Leica Microscopy Systems Ltd., Heerbrugg, Switzerland) with images collected using a Nikon Coopix 995 digital camera (Nikon Corporation, Tokyo, Japan).

Blocks of tissue including both skin and underlying outer pericarp were excised from five fruit at each stage of peeling development and fixed in either 2% paraformaldehyde and 0.1% glutaraldehyde in 0.1 M phosphate buffer (pH 7.2) (immunolabelling), or in 2% paraformaldehyde and 2.5% glutaraldehyde in 0.1 M phosphate buffer (structural observation). Samples were left under vacuum for 1 h and then either processed immediately or after 12–18 h of storage in fixative at 4 °C. Material for observation was embedded in LR White Resin (London Resin Co., Reading, UK). Tissue was washed three times in buffer, then dehydrated in an ethanol series (20%, 30%, 50%, 70%, 90%, 100% ×3) for between 15 and 20 min per step with agitation. Tissue was then infiltrated overnight with a 1:1 mix of ethanol and resin followed by three changes of pure resin over a 36 h period, and then embedded in resin in sealed moulds, polymerising overnight (around 18 h) at a temperature of 60 °C for structural observation and 55 °C for immunolabelling.

Structural observations were carried out on 1 μm sections of resin-embedded material stained in a 0.05% solution of Toluidine blue O in benzoate buffer pH 4.4 (0.125% *w*/*v* benzoic acid, 0.145% *w*/*v* sodium benzoate in water). Sections were air-dried, mounted in Shurmount (Triangle Biomedical Sciences, Durham, NC, USA), and viewed using an Olympus Vanox AHBT3 microscope (Olympus Optical Co Ltd., Tokyo, Japan). Images were collected with a Photometrics CoolSnap digital camera (Roper Scientific Ltd., Tucson, AZ, USA).

Immunolabelling was carried out using the antibodies JIM5, JIM7 and LM5 (PlantProbes, Leeds, UK) on 0.4 μm thick sections of resin-embedded tissue [51]. Sections were pretreated with PBS-T (phosphate-buffered saline plus 0.1% Tween 80) then treated with 0.1% bovine serum albumin (BSA-c; Aurion, Wageningen, The Netherlands) in PBS-T for 15 min as a blocking agent. Sections were then incubated in the appropriate antibody diluted in 0.1% BSA-c in PBS-T (JIM5 and JIM7 1:10 *v*/v, LM5 1:200 *v*/v) in a humid chamber at 4 °C overnight. Slides were washed with 2–3 mL PBS-T and sections incubated for 1 h in a humid chamber at ambient temperature with a goat anti-rat secondary antibody conjugated to Alexa™ 488 fluorescence probe (Invitrogen, Carlsbad, CA, USA) diluted 1:600 in PBS. Sections were washed with 2–3 mL of PBS-T followed by 2–3 mL of ultrapure water, then allowed to dry and mounted in Citifluor. Material was observed with a Vanox microscope and images collected with a CoolSnap camera using a fixed exposure time for each antibody.

### Extraction and analysis of cell wall material

Cell wall material was prepared from fruit material collected from two seasons. Approximately 10 g of ground frozen tissue was extracted with ethanol (final concentration 75% *v*/v) and homogenised for 2 min using a polytron. After centrifugation (15 min, 4 °C, 3000 x*g*), the supernatants were discarded. The insoluble pellets were resuspended three times in ethanol (90% *v*/v) followed by three times in acetone using a polytron. Samples were centrifuged in between washes and supernatants discarded. The final pellet was suspended in water and freeze-dried. Removal of starch was not required, as fruit were ripe.

The neutral monosaccharide content for each developmental stage, tissue, and genotype was determined by hydrolysis of two samples per season from two separate seasons (*n* = 4) in 2 M trifluoroacetic acid for 1 h at 121 °C followed by the derivatisation of the monosaccharides into alditol acetates [52]. Alditol-acetates were separated by gas–liquid chromatography using myo-inositol as an internal standard. Each sample (1 μL) was injected into a Hewlett Packard 6860 gas chromatography system equipped with a fused silica capillary column [(30 m × 0.25 mm × 0.2 μm) SP-2380, Supelco] maintained at 120 °C and fitted with a flame ionisation detector (FID) set at 240 °C. After the sample was injected, the temperature was held for 2 min, and increased 10 °C per min to 240 °C, where it was held for a further 35 min, using N_2_ as the carrier gas. Two injections were performed for each sample. Cell wall monosaccharides were identified and quantified by comparison with retention times of the monosaccharide standards, and quantities per μL injected sample calculated using Chemstation (Agilent).

Uronic acids were measured by hydrolysis of the cell wall material in 96% sulphuric acid [53] and quantified by a colourimetric reaction using the method of Blumenkrantz et al., [54] against galacturonic acid as the standard. The UA content for each developmental stage, tissue, and genotype was determined by hydrolysis of two samples per season from two separate seasons (*n* = 4), and the colour reaction carried out with three technical replicates for each sample.

The degree of pectin methylesterification (DE) was determined by gas chromatographic quantification of methanol after saponification of pectin as described in Ng et al., [55] and calculated as a molar ratio of methanol to uronic acid. The DE for each developmental stage, tissue, and genotype was determined using two samples per season from two separate seasons (*n* = 4), and three technical replicates for each sample.

### Gene expression analyses

Total RNA was extracted from kiwifruit tissue according to the method of Chang et al., [56] and pretreated with RQ1 DNase I (1 μL; Promega) to remove contaminating genomic DNA. RNA concentration was measured using a NanoDrop® ND-2000 UV-Vis Spectrophotometer (NanoDrop Technologies, Thermo Scientific, USA) at 260 nm. DNAse-treated RNA (2 μg) was used to synthesise first-strand cDNA using Superscript III (Invitrogen), and oligo d(T)20 to a total volume of 20 μl. The cDNA was diluted 1:10 with water, and 2 μL of the diluted cDNA was used as a template for RT-qPCR analysis. Reactions were performed in quadruplicate in a total volume of 10 μL, 5 μM for each primer, and 10 μL of 2× SYBR Green PCR Master Mix (Applied Biosystems) on an ABI 7500 sequence detection system (Applied Biosystems). Primer sequences are listed in Additional file 3: Table S1. The qPCR programme included a preliminary step of 10 min at 94 °C, followed by 40 cycles of 94 °C for 15 s and 60 °C for 1 min. No-template controls for each primer pair were included in each run. Kiwifruit actin was used as a reference gene [57].

### Enzyme extractions

Xyloglucan transglycosylase, xyloglucanase, xylanase, mannan transglycosylase, endo-ß-mannanase, and BGal were extracted from frozen tissue (0.5 g) ground with 50 mg polyvinylpolypyrrolidine in liquid N_2_ using a mortar and pestle. Proteins were extracted using a low salt (LS) – high salt (HS) approach, where LS buffer extracted freely soluble protein, and HS buffer extracted protein that was bound to the cell wall. Ground tissue was extracted with 1 mL LS buffer (50 mM NaCl, 10 mM dithiothreitol, 4.9 mM potassium tetrathionate, in 0.2 M sodium acetate, pH 4.7), suspended by vortexing after thawing, and centrifuged (11,000 x*g* for 10 min at 4 °C for all centrifugation steps). The pellet was resuspended in LS buffer (0.5 mL) using a vortex and centrifuged. The leftover insoluble material was re-suspended by vortexing in 1 mL HS buffer (0.6 M NaCl, 10 mM dithiothreitol, 2.4 mM potassium tetrathionate, in 0.3 M MES pH 6.0), left on ice for 30 min, and centrifuged. The pellet was resuspended by vortexing in 0.5 mL HS buffer using a vortex, left on ice for 20 min and centrifuged.

Xylan transglycosylase was extracted from 0.2 g of finely ground tissue using 1 mL buffer containing 0.1 M MES, 1.3 M NaCl, 0.2 M EDTA and protease inhibitor (Complete™, Roche) at pH 5.5 [45]. After thawing on ice, the extract was mixed, left on ice for 45 min, and centrifuged.

PG activity was extracted using LS buffer as described above. In preliminary experiments, the pellet after LS extraction was re-extracted twice using HS buffer (1.7 M NaCl, 10 mM dithiothreitol, 13 mM EDTA acid in 0.3 M MES pH 6), but no PG activity was found in gel diffusion assays even after desalting the HS extract into LS buffer using micro-concentrators (Vivaspin 500; 30 kDa molecular weight cut off, GE).

All supernatants were recovered and combined appropriately, volumes determined, and kept on ice until assayed for activity.

### Enzyme assays

Preliminary experiments were carried out using different assay pHs, extract volumes, and assay times to ensure linearity of all enzyme assays. Final assays were carried out at optimum pH, volume, and time using at least three extracts derived from bulk tissue (~30 fruit per developmental stage) collected over one season, with 3 to 5 technical replicates for each assay including appropriate controls.

#### Transglycosylase assays

Transglycosylases were assayed by their ability to attach [^3^H]oligosaccharide substrates to polysaccharide substrates. Assays and enzyme activity quantifications were carried out for XET as described in Schröder et al., [58], mannan transglycosylase assays as described in Schröder et al., [46], and xylan transglycosylase assays as described in Johnston et al., [45]. Transglycosylase activities are given as Bq of radioactivity incorporated into high molecular products per kBq of radioactive oligosaccharides supplied per g fresh weight per h.

#### Xyloglucanase and PG assays

Xyloglucanase and PG activity were assayed by gel diffusion. For plate preparation, substrates tamarind xyloglucan (Megazyme; 0.1% *w*/*v*) for xyloglucanase assays, or polygalacturonic acid (Sigma; 0.1% *w*/*v*) for PG assays, were combined with agarose (Sigma; 1% *w*/*v*) and gelatine (25 μg mL^−1^) and dissolved in McIlvaine buffer pH 5 (0.2 M Na_2_HPO_3_, 0.1 M citric acid) by boiling. The solutions were held at 75 °C while 4.5 mL per Petri dish (diameter 100 mm) were pipetted. After setting (~1 h), Petri dishes were put at 4 °C for at least 2 h or overnight to allow the gel to firm. A cork borer (3.7 mm diameter for xyloglucanase, and 2 mm for PG) was used to make holes in the gel, 15 mm apart and 15 mm from the edge. *A. eriantha* extracts (2 μL for xyloglucanase and 6 μL for PG) were inserted, in triplicate assays. Negative controls were buffer and heat-deactivated (10 min, 100 °C) enzyme extract. After diffusion of extracts into the gel, Petri dishes were covered, sealed with Parafilm®, inverted and incubated for 20 h at 28 °C.

Xyloglucanase plates were washed in McIlvaine buffer for 30 min and stained with Congo Red (Sigma; 0.2% *w*/*v* in water) for 30 min. Gels were washed in water (2 min), 96% ethanol (10 min), followed by three changes of McIlvaine buffer (20 min each). Colour was developed to a deep pink-purple over a minimum of 2 h in 1 M NaCl, with a minimum of three changes. PG plates were stained with 10 mL Ruthenium red (Fluka; 0.05% *w*/*v* in water) for 30 min to a deep pink, and destained with water until halos were visible.

To allow for quantification of PG and xyloglucanase activity, standard curves were prepared. For quantification of xyloglucanase activity, endocellulase from *Trichoderma longibrachiatum* (EC 3.2.1.4; Megazyme) in dilutions from 1 U to 0.01 U in LS or HS was prepared, and for quantification of PG, endo-PG from *Aspergillus aculeatus* (Megazyme) in dilutions of 0.1, 1.0, 2.5, 5.0, 7.5 and 10.0 mU mL^−1^ in LS buffer. Gel diffusion assays were carried out as described above.

Enzyme activity was seen as colourless to pale circles around the wells where substrate had been hydrolysed. To measure the diameter of the circles, gels were photographed, the diameter of the cleared area digitally measured (Microsoft Paint software, Microsoft Corporation, USA), and the mean radial clearance calculated. Enzyme activity was calculated by comparing the diameter of clearance with the known concentrations of xyloglucanase or PG standards and expressed as U per g fresh weight.

#### Endo-β-mannanase and xylanase activity

AZCL-galactomannan and AZCL-xylan substrates (Megazyme) were suspended in 0.3 M MES pH 5.8 (5 mg mL^−1^). To 100 μL substrate, 20 μL protein extract was added and incubated at 28 °C overnight, in triplicate reactions. Assays were stopped by the addition of 3% Tris solution (150 μL, un-pHed) after incubation, and centrifuged (10 min at 11,000 x*g*). Supernatants (150 μL) were transferred to microtitre plates and absorbance measured at 590 nm. For background reactions, Tris was added to assays prior to enzyme extract and values subtracted from values gained using active enzyme extract. Enzyme activities were expressed as relative OD at 590 nm per g fresh weight.

#### β-Galactosidase assay

BGal assays were set up in 96-well microtitre plates in triplicate with 20 μL of 1 M sodium acetate buffer pH 4.6, 15 μL water, 20 μL of LS or HS extract, and the reaction started by adding 10 μL of 2 mg mL^−1^ *p*-nitrophenyl-β-D-galactopyranoside substrate (Sigma). Reactions were incubated at 28 °C for 1 h and terminated with 80 μL of 20 mM Na_2_CO_3_. Absorbance was read at 415 nm, and enzyme activity reported as mol *p*-nitrophenol released per h per g fresh weight using a standard curve constructed with *p*-nitrophenol made up in LS or HS extraction buffer in the range of 0.01–0.20 M.

#### β-1,4-Endoglucanase activity

Azo-α-cellulose, Azo-avicel, AZCL-HC-cellulose and Azo-CM-cellulose (Megazyme) were prepared as substrates according to the manufacturer’s instructions and assayed with LS and HS extracts (prepared as described above for XET) at three different pHs (4.7, 5.3 and 5.8). Colour development in assays was measured according to the manufacturer’s instructions.

### Western blotting and antibody production

Total protein was extracted by boiling 100 mg finely ground tissue in 0.5 mL of extraction buffer (0.1 M Bis Tris, 2 M glycerol, 0.3 M sodium dodecyl sulfate, 0.2 M dithiothreitol, 0.01% Brilliant Blue G) for 10 min. After centrifugation (10 min, 11,000 x*g*), the supernatants (10 μL) were loaded onto protein gels. Protein gel electrophoreses and western blotting and staining were carried out as described previously [59], using polyclonal antibodies developed against xylanase from papaya [60], endo-β-mannanase from tomato seeds [61], polygalacturonase MdPG1 [62] and β-galactosidase from apple, and xyloglucan endotranglycoylases XTH5 and XTH7, expansin EXP3 and pectate lyase PL1 from kiwifruit. Western blots were scanned with a high resolution scanner.

Antibodies for β-gal, XTH5, XTH7, EXP3, and PL1 were raised to recombinant N-terminal His-tagged proteins expressed and purified by Ni^2+^ affinity chromatography under denaturing conditions according to manufacturer’s instructions (GE Healthcare). Purified insoluble recombinant proteins (~500 μg) were used to raise polyclonal antibodies in rabbit (AgResearch, NZ).

### Statistical analyses

‘R’ version 3.3.2 was used to perform Analysis of variance (ANOVA) to determine if the independent variables of genotype (GP, PP), stage of detachment (ND, DD, FD), and tissue type (outer pericarp, peel) contributed significant effects to the enzyme or cell wall variables presented in this manuscript. Independent variables that were found to display significant effects in the ANOVA were then subject to a posthoc Tukey’s Honest Significant Difference test to identify which pairwise comparisons displayed significant (*p* value <0.05) changes. Statistical analysis of gene expression data was similarly done using ‘R’ version 3.3.2. Pairwise student t-tests were carried out for the independent variables of genotype (GP, PP), stage of detachment (ND, DD, FD), and tissue type (outer pericarp, peel). To simplify this analysis significant (*p* value <0.05), changes in expression were only determined within each gene expression set.

## Additional files

Additional file 1: Figure S1. Toluidine blue O-stained sections of skin and hypodermal tissue from the fruit of the good-peeling (A) and poor-peeling (D) *Actinidia eriantha* genotypes. S, compressed skin layer consisting of dead cells; H1, narrow layer of thin walled hypodermal cells; H2, thicker walled more collenchyma-like hypodermal layer. Bar = 100 μm. (JPEG 1925 kb)

Additional file 2: Figure S2. Immunolabelling of good-peeling (A, C) and poor-peeling (B, D) genotypes of *Actinidia eriantha* fruit using JIM7 and LM5 at the non-detachable (ND) stage. Both antibodies were conjugated to Alexa™488 (green). Double headed arrows indicate the extent of hypodermal tissue in each genotype. Bar = 100 μm. (PPTX 1348 kb)

Additional file 3: Table S1. Primer sequences for RT-qPCR and for cloning of *PGC1* and *PGC2*. Primers for xyloglucan tranglycosylase (XTH) genes were described previously in Atkinson et al., [35]. Primers for pectate lyase gene *PL1* were described previously in Atkinson et al., [34], and primers for actin in McAtee et al., [57]. Full-length cDNA copies of the *PGC1* and *PGC2* genes were isolated from outer pericarp cDNA by PCR performed using Platinum Taq (Invitrogen) according to the manufacturer’s protocol with oligonucleotides listed. Products were cloned into pGEM-T Easy (Promega) as per the manufacturer’s protocol and multiple clones sequenced (Macrogen Inc., Korea). (PPTX 86 kb)

Additional file 4: Figure S3. Expansin (EXP) genes with medium to low expression during development of detachability in good-peeling and poor-peeling *Actinidia eriantha* genotypes. Peelability stages: ND, detachability not developed; DD, detachability developing; FD, fully detachable peel. *n* = 3 ± standard error. Expression profiles are similar to *EXP2* (*EXP1* and *EXP7*) and *EXP5* (*EXP4*) in Fig. 5. OP = outer pericarp. (JPEG 45 kb)

Additional file 5: Figure S4. Western analyses of total protein extracts from good-peeling and poor-peeling *A. eriantha* genotypes. Band patterns are highlighted with black bars in each panel. Peelability stages: ND, detachability not developed; DD detachability developing; FD, fully detachable peel. Immunoreactive bands in outer pericarp (OP) and peel are shown for β-Gal (A, B); EXP3 (C, D) and XTH7 (E, F). No bands were observed using the other antibodies. (JPEG 34 kb)

## Abbreviations

BGal: β-galactosidase
DE: Degree of methylesterification
EGase: Endo-β-1,4-glucanase
EXP: Expansin
GP: Good-peeling genotype
HG: Homogalacturonan
HS: High salt buffer
LS: Low salt buffer
PG: Endo-α-1,4-polygalacturonase
PP: Poor-peeling genotype
RT-qPCR: Reverse transcriptase quantitative PCR
XET: Xyloglucan transglycosylase
XTH: Xyloglucan transglycosylase/hydrolase
