# Supplementary figures and images for: Cell separation in kiwifruit without development of a specialised detachment zone
Source: BMC Plant Biol. 2017 May 10;17:86. doi: 10.1186/s12870-017-1034-2 (PMC5424339; doi:10.1186/s12870-017-1034-2)

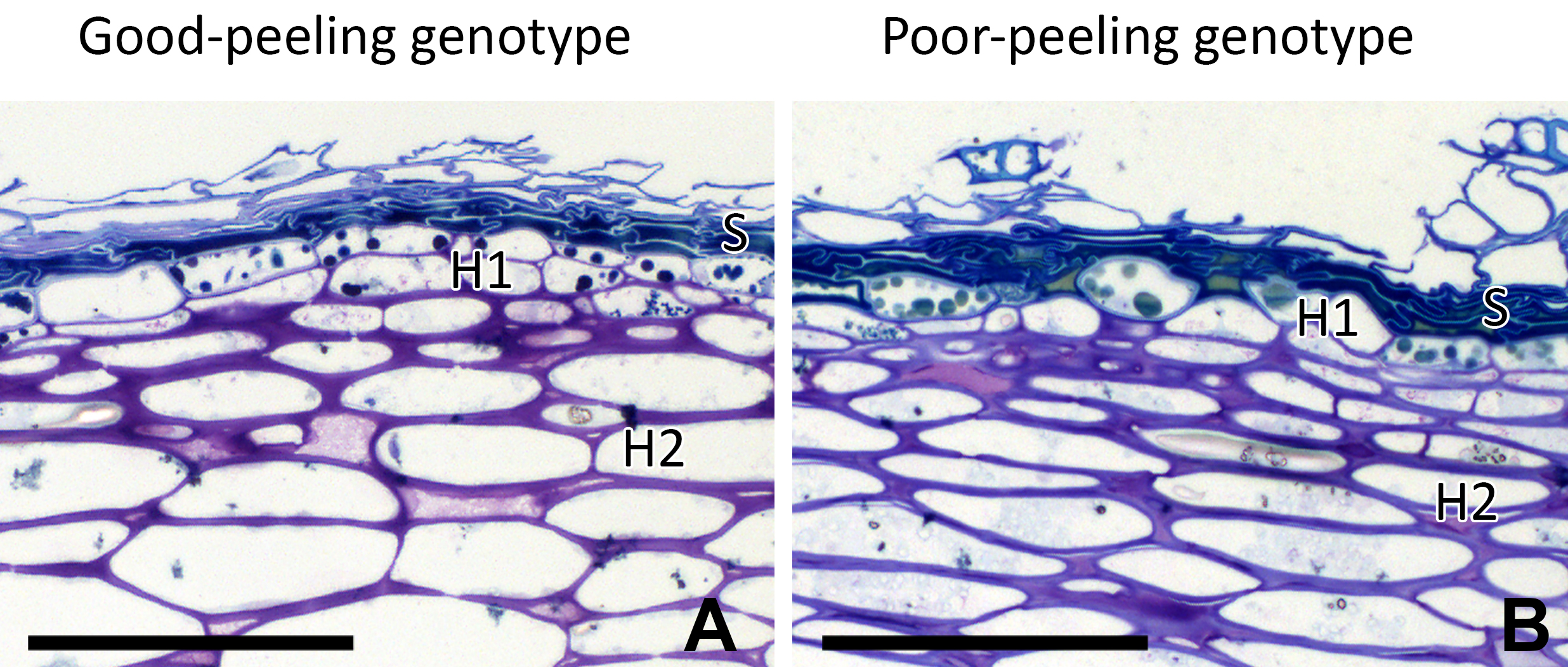

Supplement: Supplementary file 1 — Toluidine blue O-stained sections of skin and hypodermal tissue from the fruit of the good-peeling (A) and poor-peeling (D) Actinidia eriantha genotypes. S, compressed skin layer consisting of dead cells; H1, narrow layer of thin walled hypodermal cells; H2, thicker walled more collenchyma-like hypodermal layer. Bar = 100 μm. (JPEG 1925 kb) [file 12870_2017_1034_MOESM1_ESM.jpg]

## Slide 1
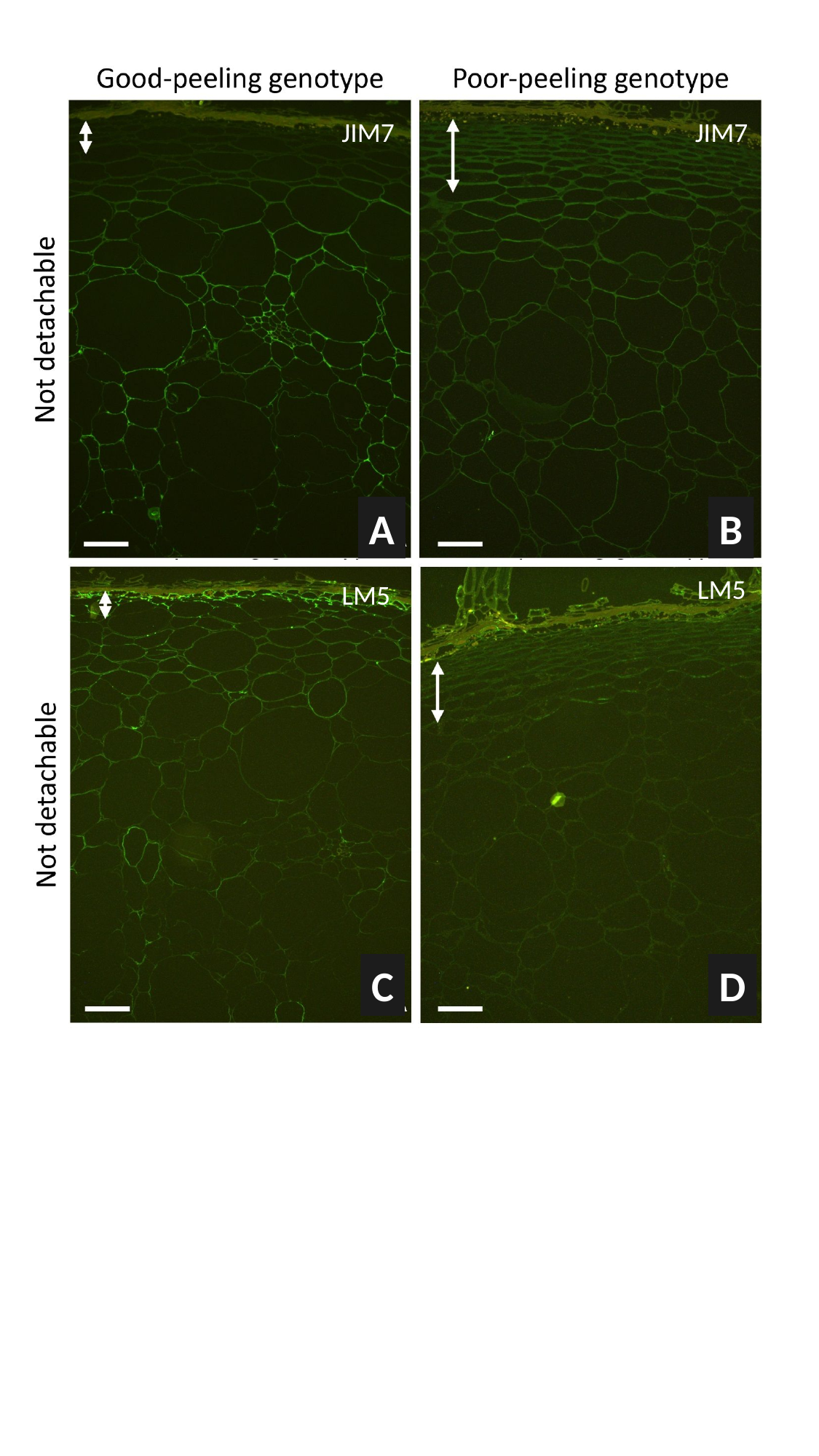

JIM7
JIM7
A
B
LM5
LM5
C
D

Supplement: Supplementary file 2 — Immunolabelling of good-peeling (A, C) and poor-peeling (B, D) genotypes of Actinidia eriantha fruit using JIM7 and LM5 at the non-detachable (ND) stage. Both antibodies were conjugated to Alexa™488 (green). Double headed arrows indicate the extent of hypodermal tissue in each genotype. Bar = 100 μm. (PPTX 1348 kb) [file 12870_2017_1034_MOESM2_ESM.pptx]

## Slide 1
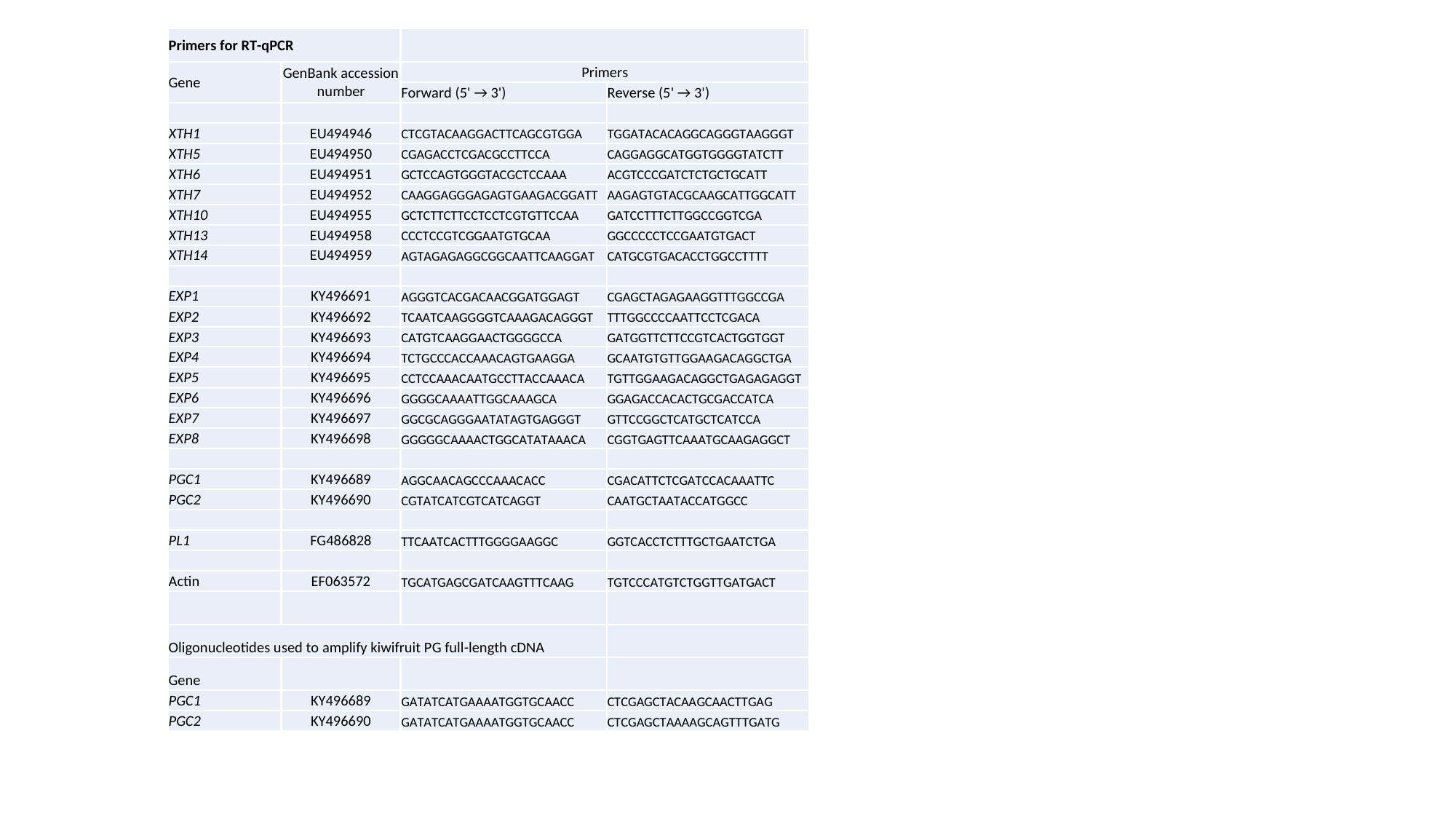

Supplement: Supplementary file 3 — Primer sequences for RT-qPCR and for cloning of PGC1 and PGC2. Primers for xyloglucan tranglycosylase (XTH) genes were described previously in Atkinson et al., [35]. Primers for pectate lyase gene PL1 were described previously in Atkinson et al., [34], and primers for actin in McAtee et al., [57]. Full-length cDNA copies of the PGC1 and PGC2 genes were isolated from outer pericarp cDNA by PCR performed using Platinum Taq (Invitrogen) according to the manufacturer’s protocol with oligonucleotides listed. Products were cloned into pGEM-T Easy (Promega) as per the manufacturer’s protocol and multiple clones sequenced (Macrogen Inc., Korea). (PPTX 86 kb) [file 12870_2017_1034_MOESM3_ESM.pptx]

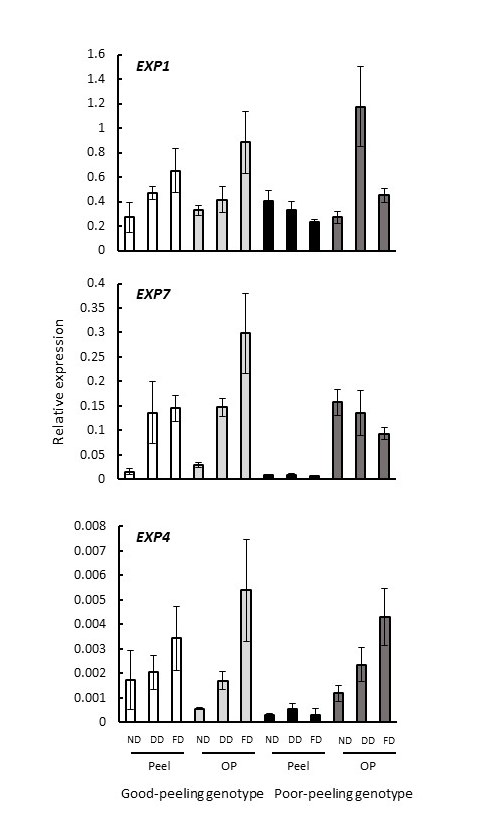

Supplement: Supplementary file 4 — Expansin (EXP) genes with medium to low expression during development of detachability in good-peeling and poor-peeling Actinidia eriantha genotypes. Peelability stages: ND, detachability not developed; DD, detachability developing; FD, fully detachable peel. n = 3 ± standard error. Expression profiles are similar to EXP2 (EXP1 and EXP7) and EXP5 (EXP4) in Fig. 5. OP = outer pericarp. (JPEG 45 kb) [file 12870_2017_1034_MOESM4_ESM.jpg]

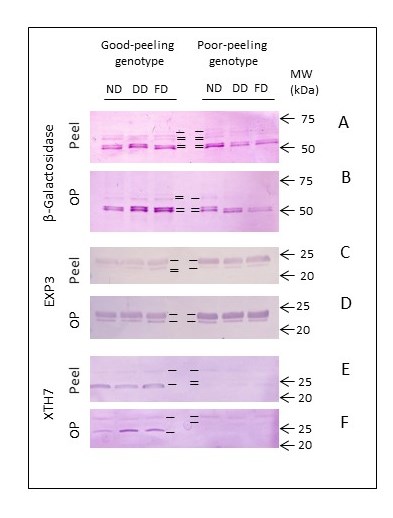

Supplement: Supplementary file 5 — Western analyses of total protein extracts from good-peeling and poor-peeling A. eriantha genotypes. Band patterns are highlighted with black bars in each panel. Peelability stages: ND, detachability not developed; DD detachability developing; FD, fully detachable peel. Immunoreactive bands in outer pericarp (OP) and peel are shown for β-Gal (A, B); EXP3 (C, D) and XTH7 (E, F). No bands were observed using the other antibodies. (JPEG 34 kb) [file 12870_2017_1034_MOESM5_ESM.jpg]
